# Supplementary figures and images for: Exploring the cytotoxic mechanisms of Pediocin PA-1 towards HeLa and HT29 cells by comparison to known bacteriocins: Microcin E492, enterocin heterodimer and Divercin V41
Source: PLoS One. 2021 Sep 2;16(9):e0251951. doi: 10.1371/journal.pone.0251951 (PMC8412286; doi:10.1371/journal.pone.0251951)

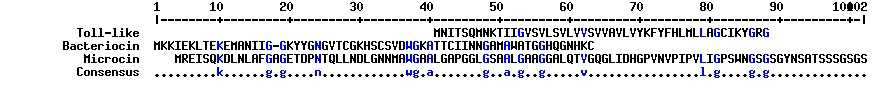

Supplement: S1 Fig — (TIF) [file pone.0251951.s001.tif]

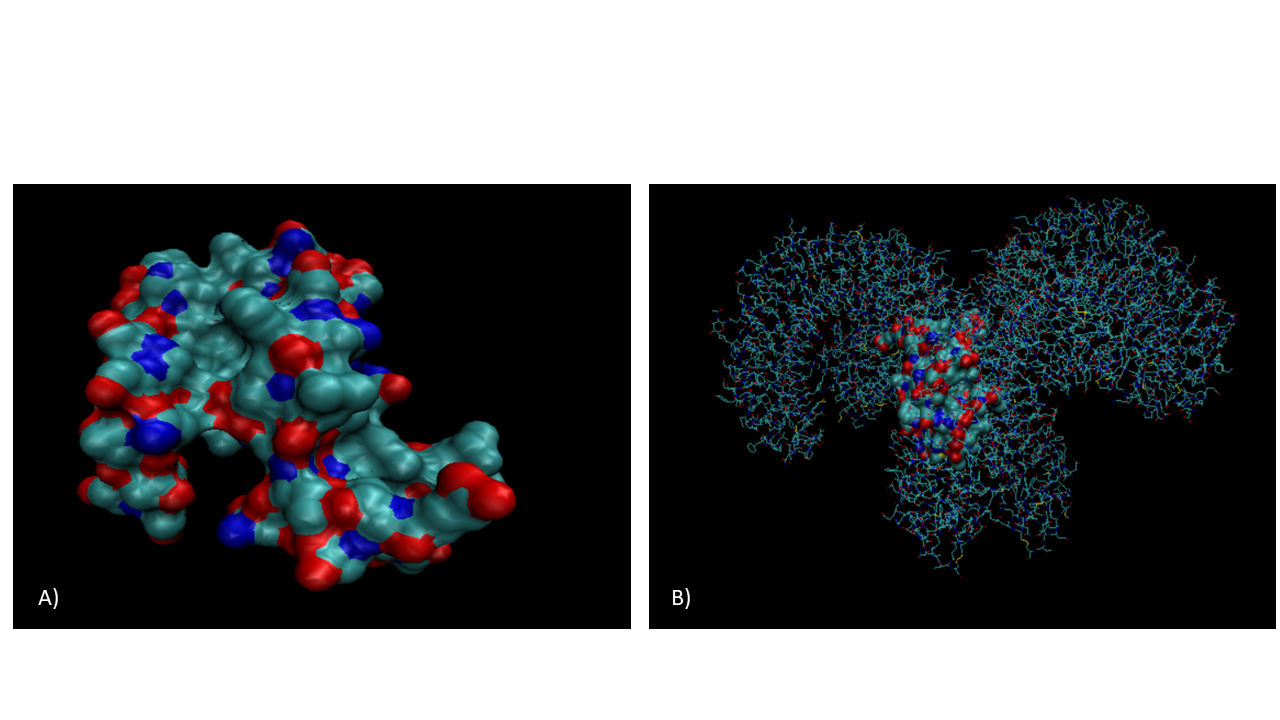

Supplement: S2 Fig — (TIF) [file pone.0251951.s002.tif]

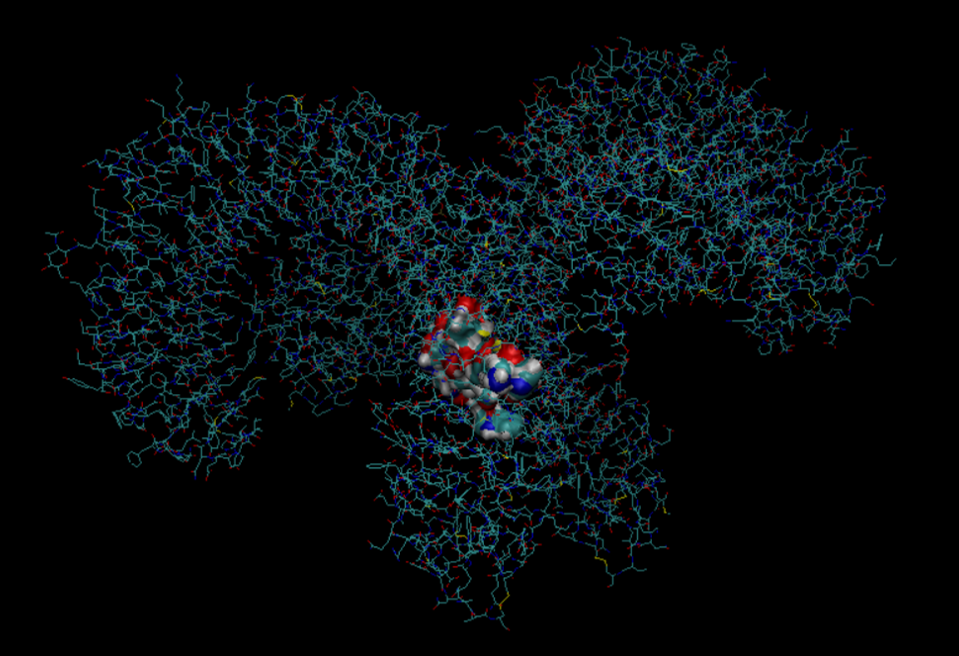

Supplement: S3 Fig — (TIF) [file pone.0251951.s003.tif]

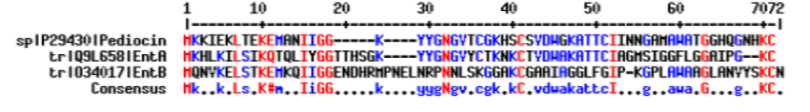

Supplement: S4 Fig — (TIF) [file pone.0251951.s004.tif]

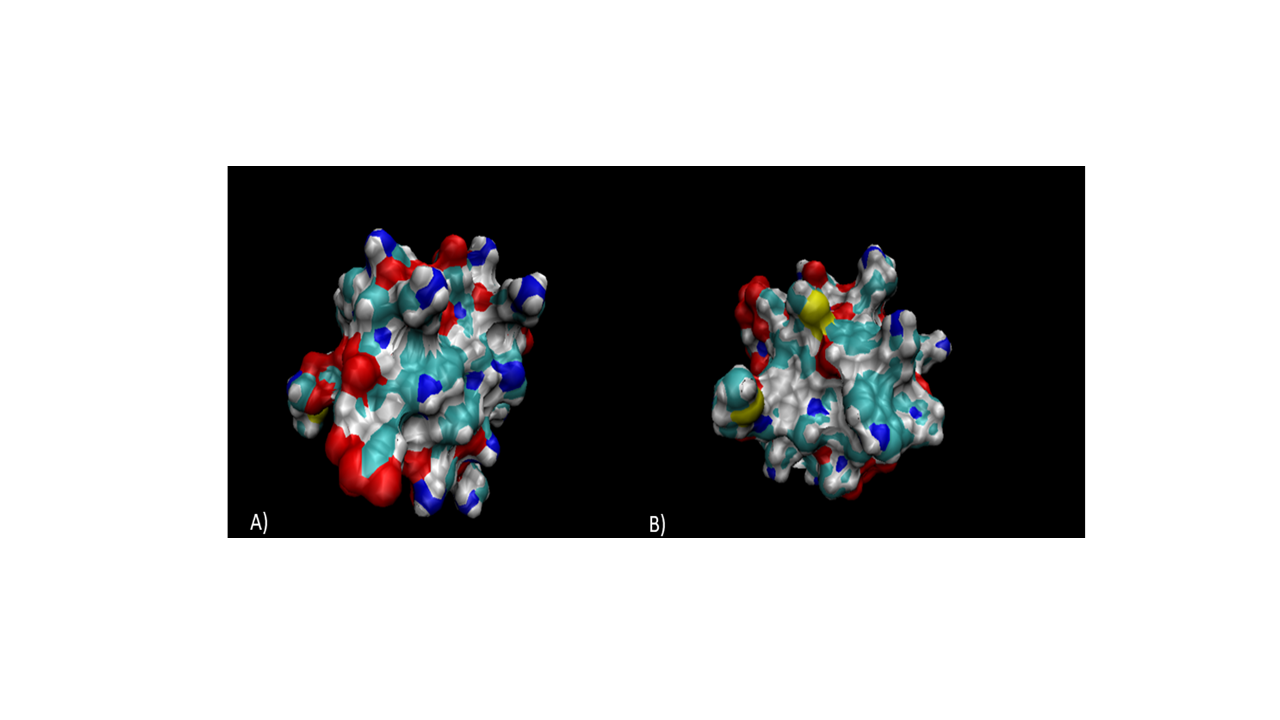

Supplement: S5 Fig — (TIF) [file pone.0251951.s005.tif]

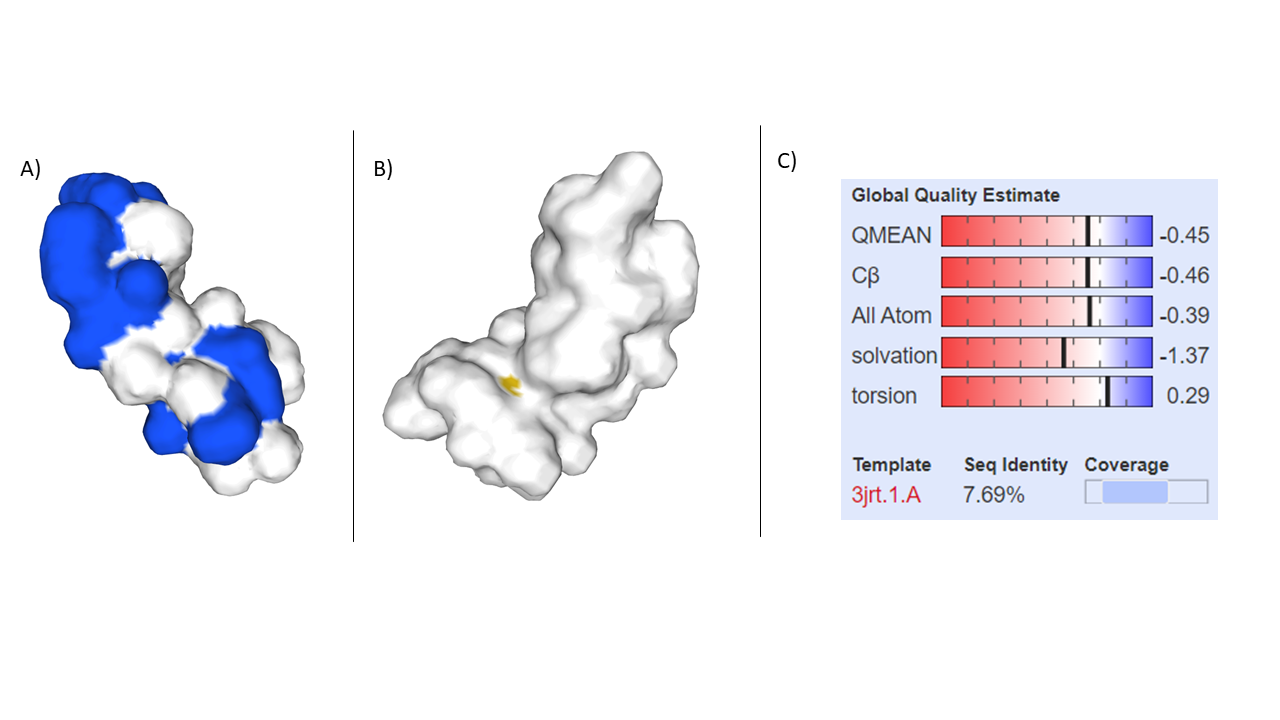

Supplement: S6 Fig — (TIF) [file pone.0251951.s006.tif]

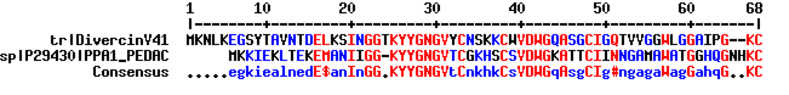

Supplement: S7 Fig — (TIF) [file pone.0251951.s007.tif]

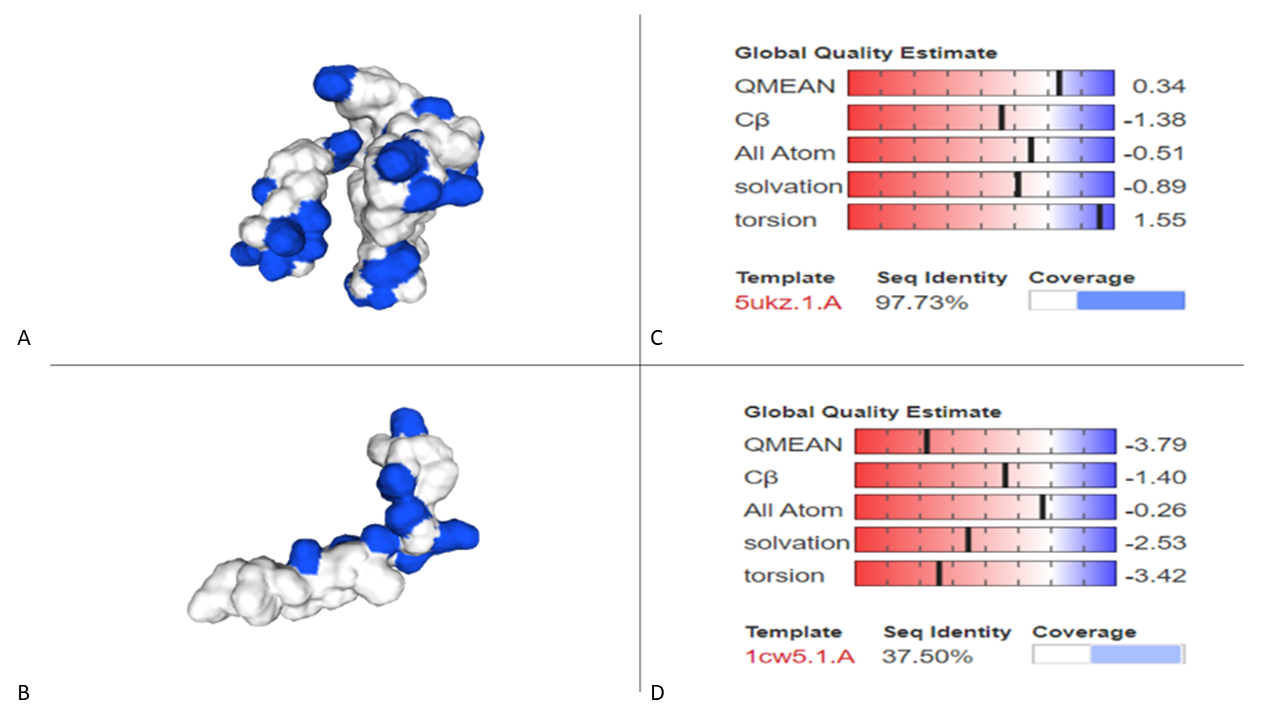

Supplement: S8 Fig — (TIF) [file pone.0251951.s008.tif]

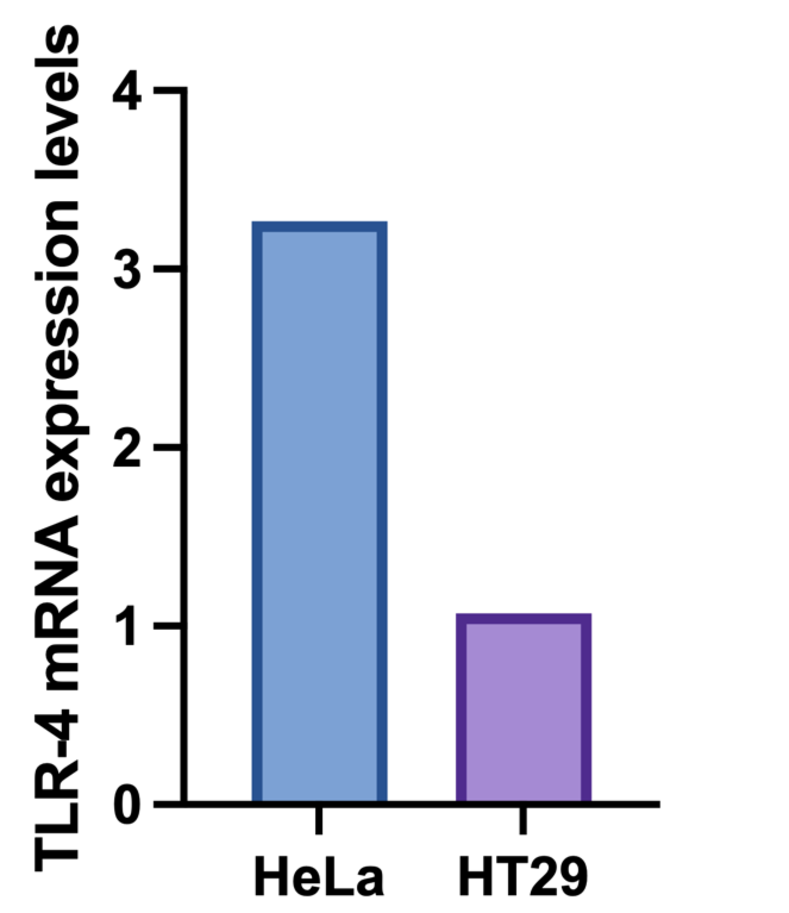

Supplement: S9 Fig — (TIF) [file pone.0251951.s009.tif]
