# Supplementary material for: Exploring the cytotoxic mechanisms of Pediocin PA-1 towards HeLa and HT29 cells by comparison to known bacteriocins: Microcin E492, enterocin heterodimer and Divercin V41
Source: PLoS One. 2021 Sep 2;16(9):e0251951. doi: 10.1371/journal.pone.0251951 (PMC8412286; doi:10.1371/journal.pone.0251951)
Supplement: Attachment — Submitted filename: Letter of Rebuttal.pdf [file pone.0251951.s011.pdf]

Dear Editor,

Firstly, we would like to thank the reviewers for their acceptance of this article with minor revisions, as well their kind and encouraging feedback. Please also find an edit to our reference list – on review we found that citations 15 and 16 were duplicates, therefore we have edited citation 16 to include the correct reference. We have also edited the title of the manuscript to match that of the online submission. In regard to references, we have checked that they match the requirements of the journal.

Please also find the addition of a ninth figure, this is in response to the second reviewers third comment.

We have made changes to most edits, however some edits we have contested. Please find below our reply to the feedback provided.

Kind Regards,

George Buss BSc (Hons)

Corresponding Author

Dr Cornelia Wilson BSc (Hons), PhD

Principal Supervisor

<u>Reviewer 1</u>

1) Abstract -
Line 33 - *"Pediocin PA-1 and Microcin E492* ***and*** *3D modelling", please replace "and" with "whereas"*

    - Please find the edit completed.

Line 39-42 - *Please revise the statement as below –*
"Unfortunately, due to the COVID-19 pandemic, we were unable to carry out experiments in the lab, and the unavailability of important data we were unable to provide and validate our solid conclusions, but rather suggestions."

    - Please find the edit completed. We have also added '..unavailability of important data **meant** we are unable…' in order to allow the sentence to read correctly.

Line 42 - , *Remove – "However", however and despite mean the same. So, please use either one.*

    - Please note the removal of *"However"*.

2) Introduction -
Line 55, please remove " ' " after diagnosis

    - We intended this as the plural for 'diagnosis', therefore we have used 'diagnoses' instead.

Line 56, replace "looks at" with "investigated"

    - Please find this edit completed.

Line 59, replace " – (Hyphen)" with a " , (comma)"

    - As this is clarifying information regarding the range of neurotoxic effects of conventional therapies we find a comma would not be appropriate, and it does not make sense in starting a new sentence. Therefore, we feel the dash mark is appropriate in this sentence.

Line 69, replace "looking into" with "discussing"

    - Please find the edit completed

Line 71, replace "researched in greater detail" with "thoroughly researched or thoroughly evaluated."

- This sentence refers to previous studies of bacteriocins carried out, so please find the edit "…which have been previously thoroughly researched…"

Line 73, remove "been"

- We find that this edit would make the sentence in question no longer make grammatical sense, and as such we disagree with this edit.

Line 78 – 80, replace with: "against HT29, despite it belonged to the same class of toxin as Pediocin PA-78 1 we hope to identify the difference between these two bacteriocins, and thereby gain greater insight into Pediocin PA-1's mechanism of action."

- Please find this edit completed.

Line 82, please replace "when it has come" with "pertaining"

- Please find this edit completed.

Line 83, please replace "the structure have been shown to form a disulphide bridge" with "the structure indicated a disulphide bridge formation"

- Please find this edit completed.

Line 84, please replace "found" with "reported"

- Please find this edit completed.

Line 86, please remove "been"

- Please find this edit completed

Line 86-87, please replace "permeabilization of the lipid bi-layer whilst also inducing apoptosis by bio-energetic collapse" with "the lipid bi-layer permeabilization whilst inducing apoptosis as well by bio-energetic collapse"

- Please find this edit completed.

Line 88, please replace "been shown" with "demonstrated"

- Please find this edit complete.

Line 90, please replace "has a" with exhibited"

- Please find this edit complete.

Line 105, please replace "In this way we were able to give a strong argument for further study, as well", with "In this way we were able to provide a fundamental understanding at molecular level for further investigation, as well"

- Please find this edit complete.

3) Methodology -

For all the softwares used in this work such as UniProt, MultiAlin and VMD, please provide the version, name of developer, country of origin, etc.

- We would like the reviewer to note that these bioinformatic tools are mainly web-based and have all been appropriately referenced, therefore all pertinent information can be identified within the bibliography.

Line 155, please replace "PBD" with "PDB"

- Please find this edit completed.

Figure 1 is actually a table, so please convert this to a table and replace in-text citation pertaining to this table.

- Please find the in-text citation completed.

4) Results Microcin E492 –

Line 193, please replace "to the best of our knowledge, the best model" with "it was the only best possible model"

- Please find this edit completed.

Line 196, please replace "come fold" with "come together to fold"

- Please find this edit completed.

5) Results – Enterocin –

Line 227, please replace "research has highlighted that Enterocin A and B potentially have different mechanisms of action" with "research has highlighted potentially different mechanisms of actions for Enterocin A and B"

- Please find this revision completed.

Line 228, please replace "by" with "upon" and "give" with "provide"

- Please find this edit completed.

Line 240-241, please replace "there were no disulphide bridges detected" with "no disulphide bridges were detected"

- Please find this edit complete.

6) Divercin V41 (Did you mean – "Results – Divercin V41",please be consistent)

- Thank you for this observation. Please find the title corrected.

Line 275, please replace "could go someway to explain why Divercin V41 does not show" with "could explain up to certain extent, the reason for Divercin 41's inability to"

- Please find this revision complete.

Line 281, please replace "on analysis" with "upon investigation"

- Please find this revision complete.

7) Discussion –

Line 308, please add a "comma" between that and when

- Please see below.

Line 309, please add a "comma" between cells and necrosis

- Please find between lines 307-310 we have rephrased the wording of this sentence to read better.

Line 315, please replace "-" with ","

- Please find this edit completed.

Line 318, please remove "-" and add a word "with" between alongside and TLR-2.

- We find that this edit does not make grammatical sense with the flow of the sentence.

Line 323 – 324, please replace "limitations of experimental data available, and the COVID-19 restrictions which prevented us from carrying out lab work we were unable to effectively look at this." With "COVID-19 restrictions the experimental data were not generated to further validate this hypothesis"

- Please find this edit completed.

Line 341-344, this whole statement is unclear, please re-phrase it for better understanding.

- Please find this rephrased slightly. This was a critique of the cited research, stating that whilst it - did observe an apoptosis effect from Enterocin A and B, it failed to go further to identify the pathway which led to apoptosis.

8) Conclusion –

Please draw a conclusion. I was hoping to see a conclusion with a summary of current computational work. Also, state how this work can be extended to future similar work.

- Please find the addition of a conclusion section in which we have summarised the findings of both the sequence alignment and 3D-modelling of all bacteriocins, highlighting the differences seen between bacteriocins which have shown a significant cytotoxic effect towards *HeLa* and/or HT29 cells, and those that have not. Please also find a summary of how these findings can feed into future studies which could ultimately have a profound effect on the way we treat patients with colon and cervical cancer.

<u>Reviewer 2</u>

1) *Page 4, Line 85: Please provide details about the respective protein receptors here.*

- Firstly, we would like to apologise to the reviewer as when we looked at the reference for citation 16 it was a duplicate of citation 15. Please find the reference list corrected. The referenced paper failed to characterise the receptors in general, instead talking about cell-surface protein receptors in general, therefore we are unable to provide details about these receptors.

2) *Page 4, Lines 84-91: These statements need further explanation. A clear reason behind the argument that "it is likely Pediocin PA-1 also has a dual mechanism of cytotoxicity" is missing here. Please elaborate.*

- Please find further clarification added – it is due to previous observations of Pediocin inducing apoptosis in competing bacterial cells without the use of protein receptors, whilst also having similar sequence and structure to Enterocin heterodimer – which are discussed later in the results section of the paper.

3) *Page 14, Lines 317-320: Authors are recommended to provide details or evidence on the TLR-4 over-expression in HeLa cells and limited expression in human colon cells.*

- Please find citations 39 and 40 evidence the over- and under-expression of TLR-4 in *HeLa* and *HT29* cells respectively. Also please find the addition of figure 9 representing this in a graph using data obtained from citation 41.

4) *Are there any Bacteriocins available for cancer therapy and how far they are successful?*

    - Please find from line 98 an edit to the statement to ready *"Whilst bacteriocins as a therapeutic agent have not proceeded past animal studies, the results of these studies clearly demonstrate how bacteriocins offer a potential advancement in the treatment of cancer"*. Results of previous studies are discussed further within the paper.

5) *It can be understood that authors were unable to carry out experiments in the lab due to the COVID-19 pandemic. However, how far can we rely on bioinformatic tools to while there is unavailability of important data? Please justify.*

    - Please find from line 42 a further clarification of how bioinformatic analysis still provides interesting and useful structural and sequence data which can be used to propose plausible and evidence-based conclusions.

6) *Conclusion needs be further clarified by the authors.*

    - As per reviewer 1, please find an additional concluding section within the paper summarising findings and the potential impact of these.
